# Expression of gemcitabine metabolizing enzymes and stromal components reveal complexities of preclinical pancreatic cancer models for therapeutic testing

**DOI:** 10.1016/j.neo.2024.101002

**Published:** 2024-05-13

**Authors:** Lisa Knoll, Jacob Hamm, Philipp Stroebel, Todorovic Jovan, Robert Goetze, Shiv Singh, Elisabeth Hessmann, Volker Ellenrieder, Christoph Ammer-Herrmenau, Albrecht Neesse

**Affiliations:** a Department of Nephrology and Hypertension, University Hospital Hannover, Carl-Neuberg-Straße 1, 30625 Hannover, Germany; b Department of Gastroenterology, Gastrointestinal Oncology and Endocrinology, University Medical Centre Goettingen, Robert-Koch-Straße 40, 37075 Goettingen, Germany; c Institute of Pathology, University Medical Center Goettingen, Goettingen, Germany; d Clinical Research Unit KFO5002, University Medical Center Goettingen, Goettingen, Germany

**Keywords:** PDAC, Gemcitabine, Chemotherapy resistance, Tumor microenvironment, Stroma

## Abstract

**Background:**

Pancreatic ductal adenocarcinoma (PDAC) poorly responds to antineoplastic agents. Discrepancies between preclinical success and clinical failure of compounds has been a continuous challenge and major obstacle in PDAC research.

**Aim:**

To investigate the association of the tumor microenvironment (TME) composition and gemcitabine metabolizing enzyme (GME) expression *in vitro* and several i*n vivo* models.

**Methods:**

mRNA expression and protein levels of GME (cytosolic 5′-nucleotidase 1 A; NT5C1A, cytidine deaminase; CDA, deoxycytidine kinase; DCK), gemcitabine transporters (ENT1, ENT2, RRM1, RRM2) and stromal components (hyaluroninc acid, podoplanin, masson trichrome, picrosirius) were assessed by qRT-PCR and immunohistochemistry in murine LSL-Kras^G12D/+^;LSL-Trp53^R172 H/+^; Pdx-1-Cre (KPC), orthotopically transplanted mice (OTM), human primary resected PDAC tissue (hPRT), corresponding patient-derived xenograft (PDX) mice, and KPC-SPARC^−/-^ mice. mRNA expression of GME was analyzed in PDAC cell lines (Panc-1, MIA PaCa, BXPC3 and L3.6) upon incubation on collagen or pancreatic stellate cell (PSC) conditioned media by qRT-PCR.

**Results:**

Endogenous KPC tumors exhibited significantly higher levels of GME compared to OTM. However, GME levels did not differ between hPRT and corresponding PDX mice. Using Kendalls Tau correlation coefficient we did not show a significant correlation of GME and components of the TME except for NT5C1A and hyaluronic acid in PDX mice (p=0.029). GME were not significantly altered upon SPARC depletion *in vivo*, and upon treatment with PSC-conditioned media or incubation on collagen plated dishes *in vitro*.

**Conclusions:**

Our findings suggest that the expression of GME is independent from the deposition of stromal components. KPC mice are most appropriate to study stromal composition whereas PDX mice maintain GME expression of the corresponding hPRT and could be best suited for pharmacokinetic studies.

## Introduction

Among all cancer entities, PDAC still has one of the lowest 5-year-survival rates with 12 %[1]. Molecular targeted therapies and immunotherapy are not efficacious in PDAC and both adjuvant and palliative treatment options are based on conventional chemotherapy [2]. PDAC is estimated to rise to the second-leading cause of cancer-related deaths by 2030 in the United States, and this fact underlines the need for novel treatment options and a better understanding of mechanisms of chemoresistance [3]. The nucleoside analogue gemcitabine is used alone or in combination with nab-paclitaxel or capecitabine for adjuvant as well as palliative treatment regimen in PDAC patients [2]. However, despite the high efficacy of gemcitabine to kill PDAC cells *in vitro*, the response rate in PDAC patients is poor [4]. Thus, it is vital to understand the precise mode of interaction of gemcitabine with tumor cells but also with the TME to overcome gemcitabine resistance.

One reason for the resistance to chemotherapy in PDAC is the profound desmoplastic stroma reaction that is characterized by a dense extracellular matrix around the actual tumor cells limiting the access of chemotherapeutic compounds to tumor cells [4]. Mechanistically, pancreatic stellate cells (PSCs), which are modified cancer associated fibroblasts (CAFs), produce excessive amounts of stroma components such as collagens or hyaluronan [4]. Moreover, the matricellular protein secreted protein acidic and rich in cysteine (SPARC) which is strongly expressed by peritumoral fibroblasts in PDAC has been associated with an activated stroma reaction in PDAC [5]. However, previous studies from our group had shown that loss of SPARC with subsequently reduced amounts of mature collagen does not affect intratumoral gemcitabine delivery [6]. PDAC cells produce a multitude of growth factors such as transforming growth factor β (TGF-β) or platelet-derived growth factor (PDGF) which activate PSCs and further stimulate production of stromal components [7]. As a result of enhanced connective tissue deposition, the TME which surrounds PDAC cells is therefore characterized by hypoxia and reduced perfusion that limits intratumoral drug delivery [4].

Besides impaired drug delivery, the expression of gemcitabine metabolizing enzymes (GME) and gemcitabine transporters determine the effectiveness of gemcitabine. As gemcitabine is hydrophilic, specific transporters are necessary to facilitate a sufficient transport of gemcitabine across the cell membrane. Notably, the expression of *human equilibrative nucleoside transporter 1 (hENT 1)* exerts a significant effect on overall survival in PDAC patients [8]. Moreover, reduced expression of the *human concentrative nucleoside transporter 1 (hCNT1)* has been shown in PDAC tissue in comparison to healthy pancreatic tissue, thus limiting access of gemcitabine to the tumor [9]. Once gemcitabine enters the tumor cell, it is phosphorylated by deoxycytidine kinase (DCK) as a first step towards its active and cytotoxic form difluorodeoxycytidine triphosphate (dFdCTP). Consequently, low expression of DCK is associated with a significant shorter median survival in PDAC patients receiving gemcitabine [10]. Gemcitabine inactivation is facilitated by cytidine deaminase (CDA) which converts gemcitabine to 2′,2′-difluorodeoxyuridine and cancer patients with lower serum levels of CDA show a significantly longer survival than patients with high CDA levels [11,4]. Moreover, a previous publication from our group had shown that overexpression of *cytosolic 5′-nucleotidase 1 A (NT5C1A)* increases gemcitabine resistance by decreasing intracellular amounts of dFdCTP [12]. Additionally, *thymidylate synthase (TYMS)* which is primarily known to inactivate 5-fluorouracil has been shown to mediate gemcitabine resistance and overexpression of TYMS was correlated with reduced survival of patients receiving gemcitabine monotherapy [13]. However, the mechanism behind this remains elusive. Summarized, the complex interplay of gemcitabine delivery/metabolization and the TME remains incompletely understood. Here, we aim to systematically characterize the association of GME and components of the TME in different model systems of pancreatic cancer in order to identify the most appropriate models for preclinical therapeutic testing.

## Materials and methods

### Human samples

Human primary resected tissue of PDAC patients was kindly made available by the Institute of Pathology of the University Medical Center Goettingen. Institutional Ethical Board approval No: 11/5/7.

### Animals

Tissues from LSL-Kras^G12D/+^; LSL-Trp53^R172 H/+^; Pdx-1-Cre (KPC), KCP-SPARC^−/−^ mice, PDX and OTM were obtained from previous studies of our group [14,15]. For generation of patient derived xenograft (PDX) mice, bulk tissue from resected PDAC specimen of the University Medical Center Goettingen was implanted subcutaneously in the flanks of female SHO-Prkdc^scid^Hr^hr^ mice for the f0-generation. As soon as the tumors reached a size of 500-800mm^3^, mice were sacrificed and tumor pieces were transplanted into nude mice to generate the f1-generation. This process was repeated for the next generations. For generation of OTM mice, C57Bl6 mice were purchased from Charles River Laboratories, Germany. 150,000 tumor cells were harvested from KPC mice, suspended in 20 μl of Dulbecco's Modified Eagle's Medium (DMEM) and 20 μl of Matrigel (VWR International, Germany) for implantation into the pancreatic tail. For surgery, mice were anesthetized with buprenorphine (10μl/g body weight) i.p. and isoflurane per inhalationem via a nose cone. Mice were kept at a 12 hour light/dark cycle and fed a gamma-irradiated diet (V1534, ssniff) *ad libitum.* All animal experiments were approved by the local animal safety review board of the University Medical Center Goettingen (animal test number 15/2057 and 17/2074) and performed according to national and international laws and policies.

### Cell culture

As medium for KPC cells, Dulbecco's DMEM with 10 % FCS and 1 % non-essential amino acids (NEAA) was used. MIA PaCa, L3.6, Panc-1, BXPC3 were cultured in DMEM with 10 % FCS. Immortalized murine PSCs from a previous study were cultured in high glucose DMEM with phenol red and supplemented with 10 % FBS [12].

### Histology and immunostaining

Mouse and human tissues were fixed with a 10 % formalin solution and embedded in paraffin. 4 μm sections were cut from the tissues and processed for H&E staining and immunohistochemistry using standard protocols as previously described [12,16]. For immunohistochemistry (IHC) the following antibodies were used: CDA (ab82346, Abcam), DCK (ab96599, Abcam), NT5C1A (Assay Biotechnology Company Inc., C15296), Podoplanin (Axxora LLC; CVL-MAB50714), HA (385911-50UG, Merck Millipore), TYMS (#9045, Cell Signaling). All antibodies were diluted 1:200 in 1 % BSA in TBST. All slides were analyzed by using Fiji imaging software (v 1.52p and 2.14.0/1.54f) as published earlier by Schindelin et al [17]. For IHC stainings of CDA, DCK, NT5C1A and TYMS, 10 pictures were taken per slide. For stainings of stromal components (HA, Masson's trichrome, pisosirius and podoplanin) 7 pictures were taken per slide using Olympus DP27 camera and the Olympus CellSens Entry 1.12 software.

### RNA isolation and qRT-PCR

For RNA isolation from 2D cell cultures, the PeqLab Gold Total RNA kit was used according to the manufacturer's instructions. RNA isolation from murine tissue was performed with Trizol as previously described [12]. RNA concentration was measured by using an INTAS nanophotometer (Intas Science Imaging). RNA was reverse transcribed to cDNA and qRT-PCR was carried out by using SYBR Select Master Mix (Applied Biosystems) according to the manufacturer's instructions on a StepOne Real-Time PCR-System (Thermo Fisher Scientific).

### RNA sequencing/single cell sequencing/microarray datasets

Log2 expression data was extracted from Maurer et al. [18], Oh et al. [19] and Moffitt et al. [5] with R2 Genomics (https://hgserver1.amc.nl/cgi-bin/r2/main.cgi?open_page=login) and exported into GraphPad Prism (v7.03).

### Statistical analyses

Kendall's Tau correlation coefficient was calculated by using Statistica (v13.3). All other biostatistical analyses were performed by using GraphPad Prism (v6.05 & 7.03). Plots were made using Statistica (v13.3) and GraphPad Prism (v6.05 & 7.03). If not indicated otherwise, Mann-Whitney U-test was used when comparing between two groups. Differences between groups were considered significant at p < 0.05 and data is presented ± standard deviation (SD).

## Results

### Gemcitabine metabolizing enzyme (GME) expression in PDAC models

**Fig. 1:**
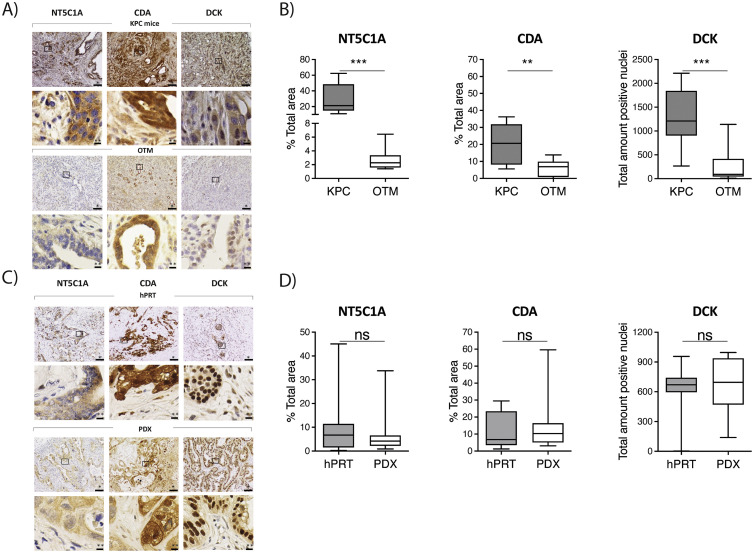
**KPC tumors exhibit a significant higher expression of GME than OTM**. A) Representative images of immunohistochemical (IHC) stainings for gemcitabine metabolizing enzymes (GME: CDA= cytidine deaminase; DCK= deoxycytidine kinase, NT5C1A = cytosolic 5′-nucleotidase 1 A) in LSL-Kras^G12D/+^; LSL-Trp53^R172 H/+^; Pdx-1-Cre (KPC) mice and orthotopically transplanted mice (OTM). Scale bars equivalent to *100μm and **20μm. B) Levels of GME in KPC mice (n=12) and OTM (n=13) as quantified by using Fiji software (v.152p). Data are presented as mean ± SD. P-value calculated by using Mann-Whitney-U-test. * p<0.05, ** p<0.01, *** p<0.001. D) Representative images of immunohistochemical (IHC) stainings for GME in human primary resected tissue (hPRT) and patient-derived xenograft (PDX) tumors. Scale bars equivalent to *100μm and **20μm. D) Levels of GME in hPRT samples (n=12) and PDX mice (n=12) as quantified by using Fiji software (v.152p). Data are presented as mean ± SD. P-value calculated by using Wilcoxon matched-pairs signed rank test. * p<0.05, ** p<0.01, *** p<0.001.

Since the selection of an appropriate model system is crucial to study gemcitabine metabolism, we initially set out to investigate the expression of GME in different *in vivo* pancreatic cancer models. First, we evaluated the expression of *NT5C1A, CDA and DCK* in KPC mice and OTM by analyzing the expression of GME in bulk pancreatic cancer tissue. Here, we were able to show significantly elevated expression of all three enzymes in KPC tumors compared to corresponding tissue of OTM (Fig. 1A & B). As the orthotopically transplanted tumor cells were generated from KPC mice, these results suggest that transplantation of tumor cells and the extra-pancreatic environment may affect GME expression. In order to further investigate the effect of transplantation on GME, we evaluated the expression of the abovementioned enzymes in hPRT and corresponding PDX tumors. Interestingly, we observed no significant differences in the expression of all three GME between hPRT and PDX (Fig. 1 C & D). Additionally, we assessed the expression of TYMS in the various in vivo model systems we used. Here, earlier studies had suggested that TYMS overexpression might contribute to gemcitabine resistance, but the mechanism behind this remained unclear [13]. Thus, we aimed to test if TYMS expression was different within the model systems we used. Here, we did not show any significant differences in TYMS expression between KPC mice and OTM (Supplementary Figure 1A). We did however find a significantly higher TYMS expression in PDX mice in comparison to hPRT (Supplementary Figure 1B).

### Cellular and acellular stromal components in PDAC models

**Fig. 2:**
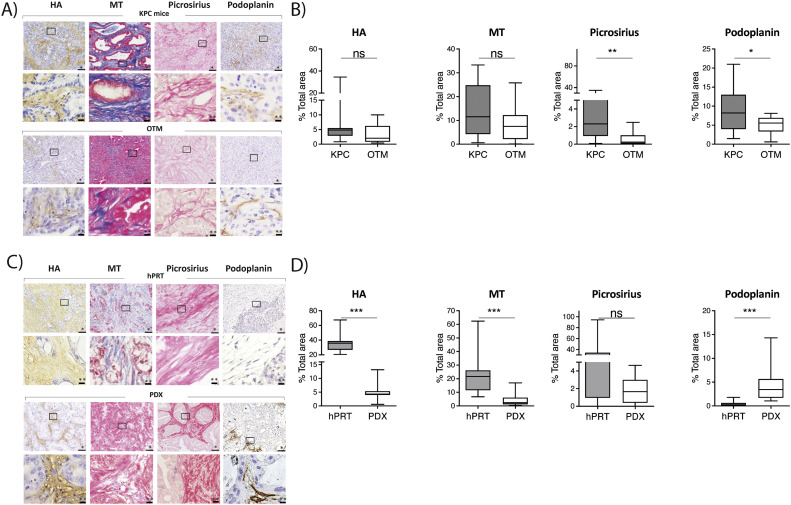
**Stromal analysis of human pancreatic tumors and various mouse models**. A) Representative images of stromal components (HA=hyaluronic acid, MT=Masson's trichrome, Picrosirius, Podoplanin) in LSL-Kras^G12D/+^; LSL-Trp53^R172 H/+^; Pdx-1-Cre (KPC) mice and orthotopically transplanted mice (OTM). Scale bars equivalent to *100μm and **20μm. B) Levels of stromal components in KPC mice (n=12) and OTM (n=13) as quantified by using Fiji software (v.152p). Data are presented as mean ± SD. P-value calculated by using Mann-Whitney-U-test. * p<0.05, ** p<0.01, *** p<0.001. C) Representative images of stromal components in human primary resected tissue (hPRT) samples and patient derived xenograft (PDX) mice. Scale bars equivalent to *100μm and **20μm. D) Levels of stromal components in hPRT samples (n=12) and PDX mice (n=12) as quantified by using Fiji software (v.152p). Data are presented as mean ± SD. P-value calculated by using Wilcoxon matched-pairs signed rank test. * p<0.05, ** p<0.01, *** p<0.001.

Since numerous previous studies had shown that the TME is implicated in therapeutic resistance in PDAC, in particular in the failure of gemcitabine [4,20], we aimed to investigate and compare the abundance of stromal components in different experimental model systems of PDAC. Here, we first set out to evaluate general stromal markers such as hyaluronic acid (HA) and collagen (picrosirius, Masson's trichrome). In addition, as the stromal components are most widely produced by CAFs, we also analyzed podoplanin as a marker for stromal fibroblasts. We initially compared stromal markers between KPC tumors and corresponding OTM and showed a significantly higher fibroblast and collagen fiber density in KPC mice compared to OTM. However, hyaluronic acid and total collagen content did not significantly differ between KPC and OTM tumor tissue (Fig. 2 A & B). These results provide evidence that murine OTM derived from KPC tumor cells harbor significantly less CAFs and collagen fibers compared to endogenous KPC tumors. Next, we tested the expression of stromal components in hPRT and corresponding PDX tumors. Here, HA and total collagen content was significantly reduced in PDX tumors compared to hPRT. However, fibroblast density (podoplanin) was significantly increased in PDX tumors compared to corresponding hPRT. (Fig. 2 C & D).

### Expression of gemcitabine metabolizing enzymes in PDAC cell lines upon collagen and PSC media stimulation

**Fig. 3:**
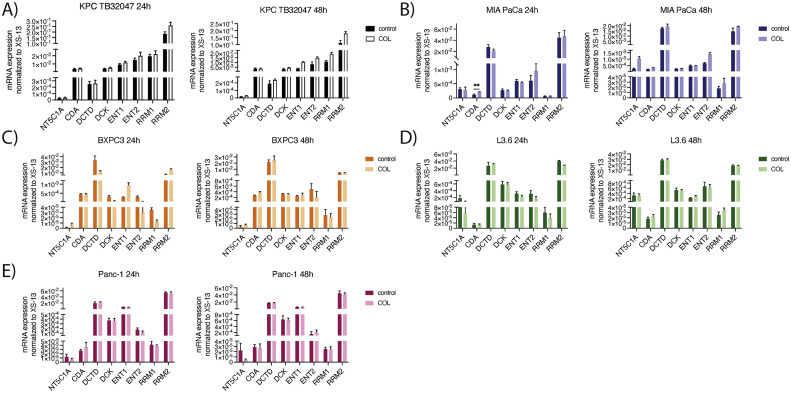
**GME expression and expression of gemcitabine transporters in various pancreatic cancer cell models is independent of collagen-I**. A-E) mRNA expression of gemcitabine metabolizing enzymes (GME) and gemcitabine transporters (CDA= cytidine deaminase; DCK= deoxycytidine kinase, NT5C1A = cytosolic 5′-nucleotidase 1 A, DCTD=deoxycytidylate deaminase, ENT1/2=equilibrative nucleoside transporter 1/2; RRM1/2=ribonucleotide reductase catalytic subunit M1/2) in murine and human pancreatic cancer cell lines (KPC TB32047, MIA PaCa, BXPC3, L3.6, Panc-1) on collagen-I coated tissue plates and controls. Data are presented as mean ± SD. P-value calculated by using Mann Whitney-U-test. * p<0.05, ** p<0.01, *** p<0.001.

In order to further delineate if specific components of the TME or soluble factors produced by CAFs affect GME and gemcitabine transporter expression in pancreatic cancer cell lines, we investigated possible effects of distinct components of the TME on GME expression. Therefore, we first set out to test the impact of collagen I on GME expression. To this end, we seeded different pancreatic cell lines onto collagen coated dishes as well as plain tissue culture plates as controls. After 24 and 48 hours, cells were harvested, RNA isolation was performed and cells were analyzed for GME and gemcitabine transporter expression by qRT-PCR. Here, we did not observe any significant differences in GME expression or expression of gemcitabine transporters between cells cultured on collagen-I-coated dishes in comparison to cells under control conditions for all different cell lines at both time-points (Fig. 3 A-E). Thus, our in vitro data suggests that the effect of collagen I on GME and gemcitabine transporters expression is negligible.

**Fig. 4:**
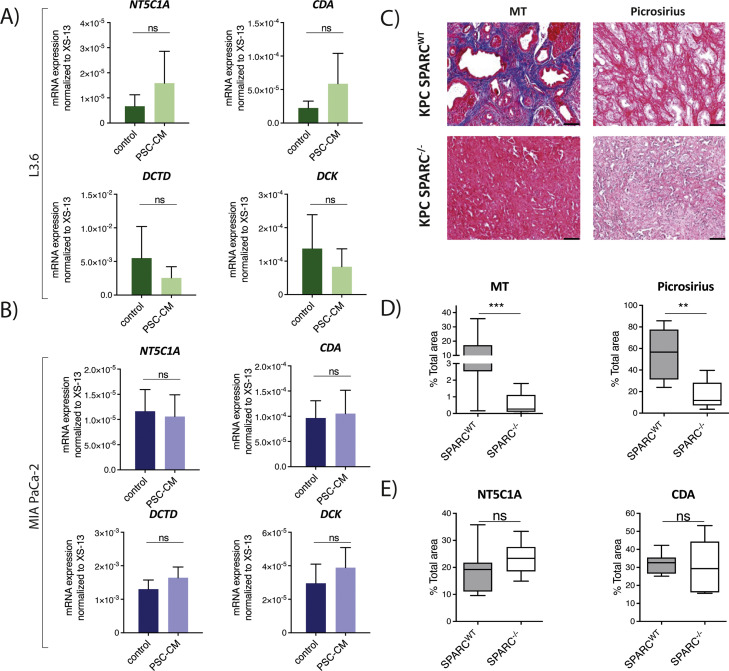
**Solid and soluble components of the TME do not significantly influence GME expression**. A-B) Expression of gemcitabine metabolizing enzymes (GME) (CDA=cytidine deaminase; DCK=deoxycytidine kinase, DCTD= deoxycytidylate deaminase; NT5C1A=5′-nucleotidase, cytosolic 1a) in L3.6 cells (top panel) and MIA PaCa-2 (bottom panel) incubated with pancreatic stellate cell conditioned medium (PSC-CM) and control medium for 48h. P-value was calculated using unpaired student-test. * p<0.05, ** p<0.01, *** p<0.001. C) Representative images of stainings of stromal components (Picrosirius, Masson's trichrome (MT)) in KPC^WT^ and KPC*^SPARC^*^-/−^ mice. Scale bars equivalent to 100μm. D) Levels of stromal components (as assessed by Picrosirius and MT=Masson's trichrome staining) in bulk tumor tissue of KPC^WT^ (n=7) and KPC*^SPARC^*^-/−^ (n=7) mice. P-value calculated by using Mann-Whitney-U-test. * p<0.05, ** p<0.01, *** p<0.001. E) Levels of GME (CDA= cytidine deaminase, NT5C1A=5′-nucleotidase, cytosolic 1a) in bulk tumor tissue of KPC^WT^ (n=7) and KPC*^SPARC^*^-/−^ (n=7) mice. P-value calculated by using Mann-Whitney-U-test. * p<0.05, ** p<0.01, *** p<0.001.

Last, we set out to test whether the changes observed in GME expression could possibly be mediated by soluble factors produced by PSCs. For this, the human pancreatic cancer cell lines L3.6 and MIA-PaCa were incubated with supernatant of PSCs and serum-free-medium as a control for 48h. RNA was isolated and qRT-PCR for GME was performed to compare GME expression between cells stimulated with supernatant of PSCs and control medium. Here, we did not observe any differences in GME expression dependent on stimulation with supernatant of PSCs (Fig. 4 A&B). In sum, we did not find a consistent and biologically relevant regulation of GME mRNA expression by collagen and PSC-derived soluble factors *in vitro* and *in vivo*.

### Correlation of stromal components and GME expression in PDAC models

To validate our findings *in vivo* and address the question whether the differences observed in GME expression between tumors of murine and human origin could be due to the observed differences in the TME upon transplantation, we aimed to test associations between GME and stromal components using the Kendall rank correlation coefficient. Apart from NT5C1A and HA in PDX mice, we did not observe any other significant correlation between GME and stromal components in KPC, OTM and hPRT (Table 1). To further explore the potential association of GME expression and collagen deposition *in vivo*, we used a KPC mouse model with a germline knockout for *SPARC* (KPC*^SPARC^*^-^^-^). Genetic deletion of SPARC in pancreatic tumors results in reduction of collagen deposition and collagen fiber density without affecting PDAC progression, vessel density, tumor incidence, grading or metastatic frequency [6]. We confirmed lower levels of overall and mature collagen in bulk pancreatic tumors of KPC*^SPARC^*^-/−^ mice compared to wildtype littermates (Fig. 4C & D). In line with the *in vitro* results, we did not observe any significant differences in expression of GME between KPC*^SPARC^*^-/^^-^ and KPC^WT^ mice (Fig. 4E). Moreover, we did not show any significant differences in TYMS expression between KPC^SPARC-/^^-^ and KPC^WT^ mice (Supplementary Figure 1C). Thus, we conclude that SPARC dependent collagen deposition does not affect expression of GME or TYMS. Eventually, we aimed to assess published human datasets comparing between tumor and stroma subsets of surgically resected PDAC specimen as well as healthy pancreatic tissue for the expression of GME to put our results in perspective. Here, we observed a significantly higher expression of CDA and a trend towards higher expression of DCK in PDAC tissue in comparison to the surrounding stroma (Supplementary Figure 2A). Moreover, we observed a higher expression of the GME CDA and DCK in bulk PDAC tissue in comparison to normal pancreatic tissue except for NT5C1A which was expressed higher in normal pancreatic tissue (Supplementary Figure 2B&C).

**Table 1:** Correlation between GME and stromal components in pancreatic cancer in vivo models.

		HA		MT		Picrosirius		Podoplanin	
	GME	τ	p	τ	p	τ	p	τ	p
KPC	NT5C1A	-0.212	0.337	-0.273	0.217	-0.333	0.131	-0.182	0.411
	CDA	-0.333	0.131	-0.030	0.900	0.212	0.337	0.364	0.100
	DCK	-0.182	0.411	-0.243	0.273	-0.243	0.273	-0.273	0.217
OTM	NT5C1A	0.103	0.635	-0.231	0.272	-0.026	0.903	-0.282	0.180
	CDA	0.026	0.903	-0.051	0.807	0.000	1.000	-0.103	0.625
	DCK	0.000	1.000	-0.026	0.903	-0.180	0.393	-0.026	0.903
hPRT	NT5C1A	0.000	1.000	0.152	0.493	-0.030	0.891	0.091	0.681
	CDA	-0.394	0.075	0.364	0.100	0.061	0.784	-0.061	0.784
	DCK	0.152	0.493	0.000	1.000	-0.182	0.411	-0.121	0.584
PDX	NT5C1A	-0.485	0.029	-0.303	0.170	-0.242	0.273	-0.212	0.337
	CDA	-0.364	0.100	-0.182	0.411	0.242	0.273	-0.212	0.337
	DCK	-0.091	0.681	-0.152	0.493	-0.030	0.891	0.121	0.584

Correlation between the levels of gemcitabine metabolizing enzymes (GME: CDA= cytidine deaminase; DCK= deoxycytidine kinase, NT5C1A = cytosolic 5′-nucleotidase 1 A) and stromal components (HA=hyaluronic acid, MT=Masson's trichrome, Picrosirius, Podoplanin) in immunohistochemistry stainings of bulk tumor tissue in LSL-Kras^G12D/+^; LSL-Trp53^R172 H/+^; Pdx-1-Cre (KPC) (n=13) and patient-derived xenograft (PDX) mice (n=12), orthotopically transplanted mice (OTM) (n=13) as well as human primary resected tissue (hPRT) (n=12) as quantified by using Fiji software (v.152p). Kendall's Tau correlation coefficient was used for calculation of p-values.

## Discussion

To the best of our knowledge, the expression of GME has not been systematically and comparatively assessed for pancreatic cancer mouse models examined in this study (OTM, KPC, PDX). Here, we provide first evidence of a significant lower expression of GME in OTM compared to KPC mice. As previous publications had shown that tumors from KPC mice are mostly resistant to gemcitabine treatment, our results therefore suggest that overexpression of GME such as *NT5C1A and CDA* could be responsible for this effect [14,21]. In addition, we show for the first time that PDX mice maintain the expression of GME compared to the corresponding hPRT suggesting that PDX tumors are the most appropriate model to study GME in regard to the pharmacokinetic and pharmacodynamic effects of gemcitabine in pancreatic cancer *in vivo*. Moreover, we assessed the expression of TYMS in KPC mice, PDX mice, OTM and hPRT since TYMS has been associated with gemcitabine resistance. Here, we show that TYMS expression does not differ between KPC mice and OTM but revealed enhanced levels in PDX mice compared to hPRT. Further studies are therefore warranted to elucidate exact mechanisms of action of TYMS and subsequent preclinical and clinical implications. Since OTMs are generated by transplantation of KPC cells and PDX are derived from transplantation of small human tissue fragments, we hypothesized that the surrounding TME could be responsible for the observed differences in GME expression. In order to test this hypothesis, we assessed if we could observe an association between solid and soluble components of the TME and GME expression *in vitro* and *in vivo*.

First, we tested the impact of collagen I on GME expression in various *in vitro* models of pancreatic cancer. Here, we did not observe any significant effects of collagen I exposure on GME expression and expression of gemcitabine transporters in the various pancreatic cancer cell lines we tested. Our in vitro data therefore suggests that GME expression is independent of contact with collagen I by pancreatic cancer cells. In addition, we examined if soluble components produced by PSCs could regulate GME. Here, we did not find any differences in GME in MIA PaCa or L3.6 cells treated with conditioned media of PSCs. Thus, regulation of GME appears to be unaffected by humoral components produced by PSCs *in vitro*. In conclusion, our results underline the limitations of *in vitro*-models to study the influence of gemcitabine treatment on pancreatic cancer cells.

*In vivo*, we assessed potential associations between *NT5C1A, CDA* as well as *DCK* and the stromal components hyaluronic acid, collagen I and podoplanin as a marker for CAFs by using the Kendall rank correlation coefficient. Interestingly, we only showed a significant correlation between HA and *NT5C1A* in PDX mice but not in hPRT, OTM or KPC mice. HA had been previously investigated as a potential therapeutic target, and the combination of human recombinant hyaluronidase (PEGPH20) and gemcitabine decreased metastatic tumor burden and increased survival in KPC mice [22,23]. However, phase II/III trials showed inconsistent results of application of PEGPH20 in combination with current standard regimes of chemotherapy (FOLFIRINOX, Gemcitabine/Nab-Paclitaxel) in patients with metastatic PDAC[[24], [25], [26]]. For combination with gemcitabine alone, the initial HALO-2 phase II trial reported an increased progression free survival [24]. However, these findings could not be replicated in a phase III trial [26]. The absence of a significant correlation of stromal components (especially HA) and GME we observed within the hPRT could thus in part explain the failed translation of the previous preclinical results regarding optimization of gemcitabine treatment with a combination with PEGPH20.

**Fig. 5:**
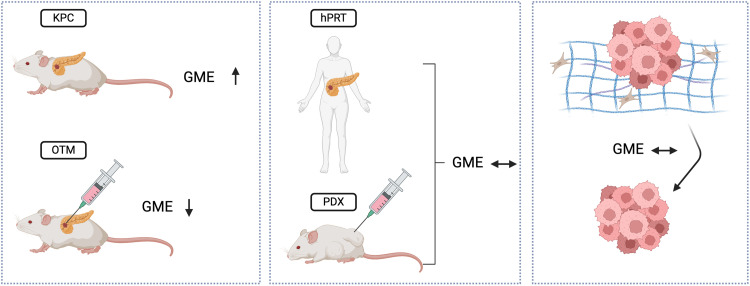
**Summarized schematic figure of the key findings of this study**. Pancreatic cancer tissue of LSL-Kras^G12D/+^; LSL-Trp53^R172 H/+^; Pdx-1-Cre (KPC) mice exhibits a significantly higher expression of gemcitabine metabolizing enzymes (GME) than orthotopically transplanted mice (OTM) whereas the expression of GME does not differ between tumor tissue within patient-derived xenograft (PDX) mice and corresponding human primary resected tissue (hPRT). Moreover, expression of GME is independent of solid/soluble stroma components in the various model systems we tested. Figure created with BioRender.com.

To additionally validate our results *in vivo,* in particular regarding the effects of collagen, we assessed GME and TYMS expression in KPC*^SPARC^*^-/^^-^ and KPC^WT^ mice as an earlier publication from our group had shown that KPC*^SPARC^*^-/−^ display reduced levels of mature collagen I. Interestingly, GME and TYMS expression did not change upon genetic ablation of SPARC in KPC mice providing evidence that the amount of mature collagen deposition in PDAC does not have implications for gemcitabine delivery or metabolization. Lastly, we investigated the expression of GME in published sequencing/microarray datasets comparing human PDAC, stroma and normal pancreas samples. Here, we found that CDA and DCK are significantly higher expressed within PDAC tissue in comparison to normal pancreatic tissue. Only for NT5C1A we observed a higher expression in normal pancreatic tissue compared to tumor tissue which could be explained by different expression of RNA and protein levels caused by post-transcriptional modifications. Moreover, we observed a significantly higher expression of CDA and a trend towards higher expression of DCK in human tumor samples in comparison to the surrounding stroma (Fig. 5).

## Conclusion

In sum, we show that KPC mice exhibit a significantly higher expression of GME in comparison to OTM mice. However, we did not observe any differences in GME expression between hPRT and tumors of PDX mice. Therefore, PDX mice adequately recapitulate GME expression in the primary tumor and display a good model system to study gemcitabine metabolism *in vivo*. Nevertheless, our data does not point towards a clear role of either solid or soluble components of the TME in modulation of GME expression *in vivo*. Therefore, indirect targeting of GME by treatment strategies altering the stromal composition of pancreatic cancer seems not to be a feasible approach. However, as the expression of GME and the abundance of stromal components seem to be independent from one another, dual targeting of both GME and TME components could be a novel approach to enhance gemcitabine treatment efficiency in future studies.

## Funding

The work was funded by the 10.13039/501100005972German Cancer Aid (to Elisabeth Hessmann: 70112108 and 70112505, to Albrecht Neesse: 70113213 [Max Eder group], to Shiv K. Singh: 70115054 [Max Eder group]); the 10.13039/501100001659Deutsche Forschungsgemeinschaft (DFG) (to Philipp Stroebel, Albrecht Neesse, Shiv K. Sing, Volker Ellenrieder, and Elisabeth Hessmann: KFO5002); Christoph Ammer-Herrmenau received startup funding from DFG (KFO5002), and Todorovic Jovan was funded by a GEROK position (KFO5002).

## CRediT authorship contribution statement

**Lisa Knoll:** Writing – review & editing, Writing – original draft, Visualization, Validation, Software, Resources, Methodology, Investigation, Formal analysis, Data curation. **Jacob Hamm:** Writing – review & editing, Writing – original draft, Visualization, Validation, Resources, Methodology, Investigation, Formal analysis, Data curation. **Philipp Stroebel:** Writing – review & editing, Resources. **Todorovic Jovan:** Writing – review & editing, Investigation, Formal analysis, Data curation. **Robert Goetze:** Writing – review & editing, Validation, Methodology, Investigation, Formal analysis, Data curation. **Shiv Singh:** Writing – review & editing, Funding acquisition. **Elisabeth Hessmann:** Writing – review & editing, Funding acquisition. **Volker Ellenrieder:** Writing – review & editing, Funding acquisition. **Christoph Ammer-Herrmenau:** Writing – review & editing, Validation, Methodology, Investigation, Funding acquisition, Formal analysis, Data curation. **Albrecht Neesse:** Writing – review & editing, Writing – original draft, Validation, Supervision, Resources, Project administration, Methodology, Funding acquisition, Formal analysis, Conceptualization.

## Declaration of competing interest

The authors declare that they have no known competing financial interests or personal relationships that could have appeared to influence the work reported in this paper.

## Appendix Supplementary materials

Image, application 1
