# Supplementary material for: Expression of gemcitabine metabolizing enzymes and stromal components reveal complexities of preclinical pancreatic cancer models for therapeutic testing
Source: Neoplasia. 2024 May 13;53:101002. doi: 10.1016/j.neo.2024.101002 (PMC11109879; doi:10.1016/j.neo.2024.101002)
Supplement: Supplementary file 1 [file mmc1.docx]

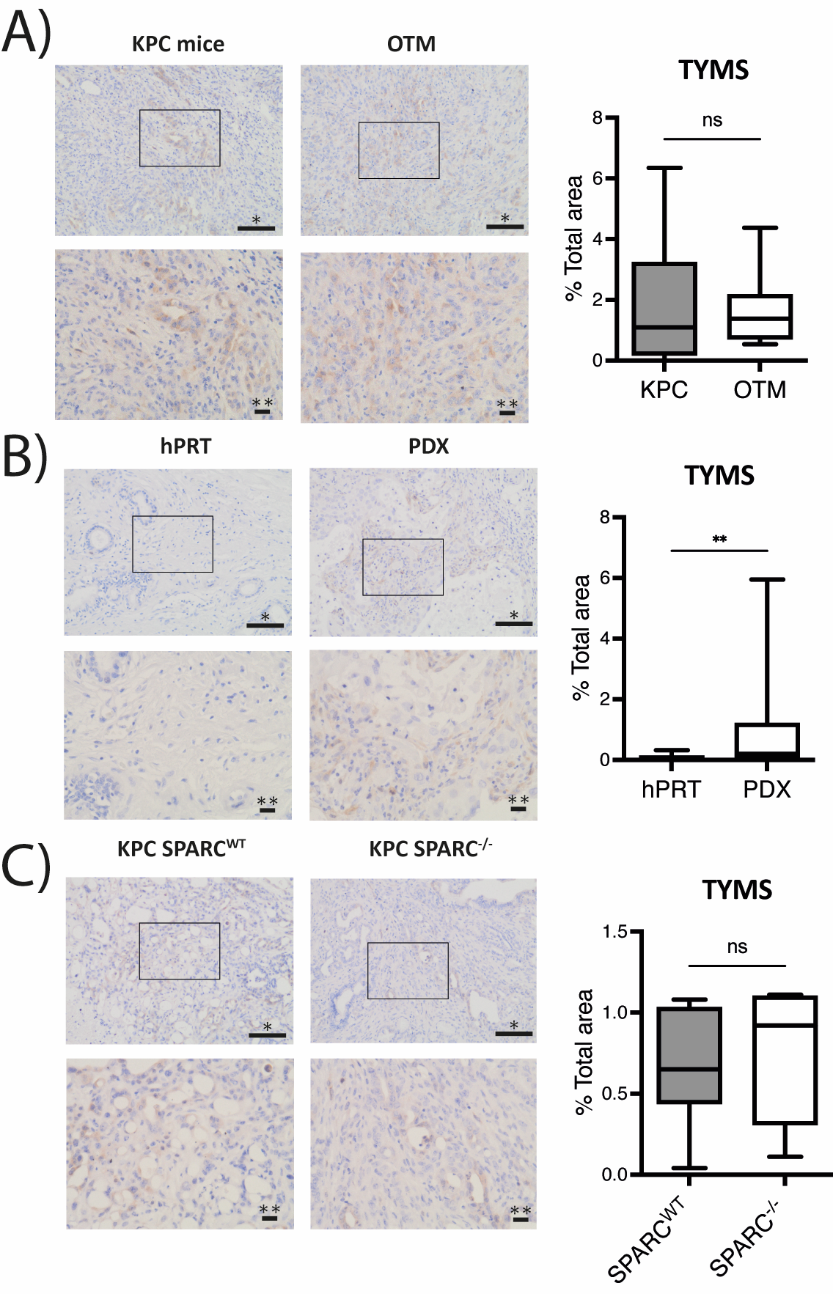


**Supplementary Figure 1 TYMS expression in various in vivo models of pancreatic cancer**

1. Percentage of total stained area and representative images of thymidylate synthase (TYMS) as assessed by immunohistochemistry staining of pancreatic tumor tissue of LSL-Kras^G12D/+^; LSL-Trp53^R172 H/+^; Pdx-1-Cre (KPC) (n=12) and orthotopically transplanted mice (OTM) (n=13).
2. Percentage of total stained area and representative images of thymidylate synthase (TYMS) as assessed by immunohistochemistry staining of human primary resected tissue (hPRT) (n=9) and pancreatic tumor tissue of corresponding patient-derived xenograft (PDX) mice (n =13).
3. Percentage of total stained area and representative images of thymidylate synthase (TYMS) as assessed by immunohistochemistry staining of pancreatic tumor tissue of KPC^WT^ (n=7) and KPC*^SPARC^*^-/-^ (n=7) mice.

Data are presented as mean ± SD. P-value calculated by using Mann-Whitney-U-test. * p<0.05, ** p<0.01, *** p<0.001. Scale bars equivalent to *100μm and **20μm.


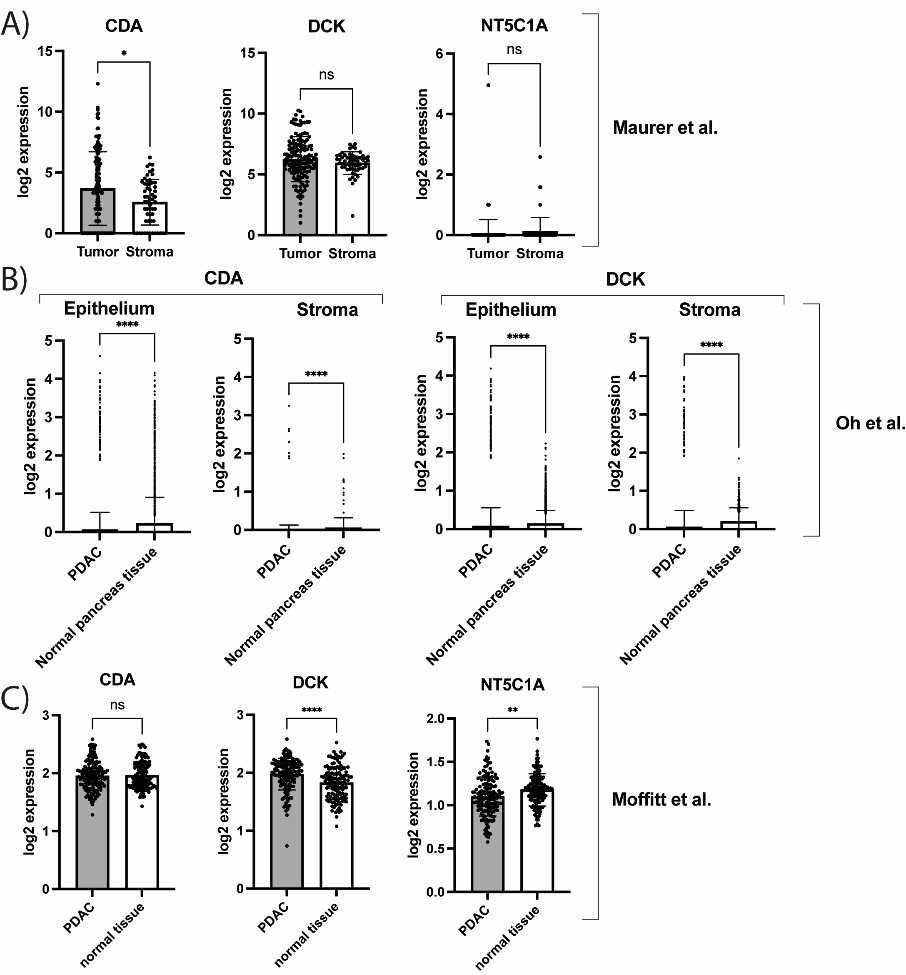


**Supplementary Figure 2 GME expression in human PDAC cohorts**

A-C) Log2 expression of gemcitabine metabolizing enzymes (GME: CDA= cytidine deaminase; DCK= deoxycytidine kinase, NT5C1A = cytosolic 5’-nucleotidase 1 A) in three independent human cohorts of PDAC patients. A) RNA sequencing data of laser capture microdissected stroma/pancreatic cancer tissue (data extracted from Maurer et al.)^19^ B) Single cell RNA sequencing data divided by stroma and epithelial fraction of pancreatic cancer tissue (data extracted from Oh et al.)^20^ Expression data for NT5C1A was not available in this dataset and could therefore not be analyzed. C) Microarray data of bulk pancreatic tissue divided into tumor tissue and adjacent normal pancreatic tissue by *in silico* “virtual microdissection” (data extracted from Moffitt et al.)^21^. Data are presented as mean ± SD. P-value calculated by Mann-Whitney-U-test. * p<0.05, ** p<0.01, *** p<0.001, **** p<0.0001.
